# Testing for Coccidioidomycosis among Patients with Community-Acquired Pneumonia

**DOI:** 10.3201/eid1407.070832

**Published:** 2008-07

**Authors:** Douglas C. Chang, Shoana Anderson, Kathleen Wannemuehler, David M. Engelthaler, Laura Erhart, Rebecca H. Sunenshine, Lauren A. Burwell, Benjamin J. Park

**Affiliations:** * Centers for Disease Control and Prevention, Atlanta, Georgia, USA; † Arizona Department of Health Services, Phoenix, Arizona, USA

**Keywords:** Coccidioidomycosis, pneumonia, serology, research

## Abstract

Coccidioidomycosis is a common cause of community-acquired pneumonia (CAP) in disease-endemic areas. Because testing rates influence interpretation of reportable-disease data and quality of CAP patient care, we determined the proportion of CAP patients who were tested for *Coccidioides* spp., identified testing predictors, and determined the proportion of tested patients who had positive coccidioidomycosis results. Cohort studies to determine the proportion of ambulatory CAP patients who were tested in 2 healthcare systems in metropolitan Phoenix found testing rates of 2% and 13%. A case-control study identified significant predictors of testing to be age >18 years, rash, chest pain, and symptoms for >14 days. Serologic testing confirmed coccidioidomycosis in 9 (15%) of 60 tested patients, suggesting that the proportion of CAP caused by coccidioidomycosis was substantial. However, because *Coccidioides* spp. testing among CAP patients was infrequent, reportable-disease data, which rely on positive diagnostic test results, greatly underestimate the true disease prevalence.

## Abstract 2

Lack of testing may lead to underdiagnosis and underestimates of disease prevalence.

Coccidioidomycosis (valley fever) is a disease caused by *Coccidioides* spp.*,* dimorphic fungi that thrive in the alkaline soil of warm, arid climates (*1*). Infection may occur when conidia in disrupted soil are inhaled. Coccidioidomycosis-endemic areas include the southwestern United States, parts of Mexico, and Central and South America. In the United States, these areas include parts of Arizona, California, New Mexico, Nevada, Texas, and Utah (*1*).

The clinical manifestations of coccidioidomycosis have been well established (*2*–*4*); 1–3 weeks after a person inhales the spores, most persons with symptomatic infection will have a clinical syndrome consistent with community-acquired pneumonia (CAP) (*3*). Serologic testing is the most frequently used method for diagnosis of primary pulmonary coccidioidomycosis (*3*,*5*). For most patients, serologic reactivity ends after a few months unless infection is active.

Although 95% of symptomatic pulmonary infections are self-limiting and resolve after several weeks or months without antifungal therapy, ≈5% progress to asymptomatic residua, such as pulmonary nodules or cavities (*2*). Among all recognized infections, extrapulmonary disease involving the meninges, bones and joints, skin, and soft tissues occurs in <5% (*2*). Risk factors for severe or disseminated infection include immunosuppression, diabetes, preexisting cardiopulmonary disease, second- or third-trimester pregnancy, and African or Filipino descent (*6*).

Previous studies suggest that the true prevalence of coccicioidomycosis is substantially underestimated (*6*,*7*). One study, which used rates of skin-test conversion, estimated that clinical illness would develop in ≈30,000 persons per year in southern Arizona (*7*). Another study found that coccidioidomycosis was serologically confirmed for 29% of CAP patients in primary care clinics in Tucson, Arizona (*8*). To understand more about the unmeasured prevalence of disease, we evaluated *Coccidioides* spp.testing practices for ambulatory clinic patients with CAP in Maricopa County, which encompasses most of metropolitan Phoenix in Arizona, a state where coccidioidomycosis is reportable. Our objectives were to estimate the proportion of patients with CAP who were tested for coccidioidomycosis, to determine predictors of testing, and to determine the proportion of CAP patients who had coccidioidomycosis. To accomplish our objectives, we performed 3 related studies: a data analysis, a retrospective cohort study, and a case–control study.

## Methods

### Descriptive Epidemiology

To describe the epidemiology of coccidioidomycosis in metropolitan Phoenix, we analyzed data from the National Electronic Telecommunication System for Surveillance. We calculated county-specific and age group–specific incidence rates for 1999–2004. Population denominators were obtained from the US Census Bureau (http://quickfacts.census.gov/qfd/index.html).

### Retrospective Cohort Studies

To determine the proportions of CAP patients tested for *Coccidioides* spp., we performed retrospective cohort studies in 2 healthcare systems (systems A and B) in metropolitan Phoenix. System A served large numbers of patients without private insurance, whereas system B was associated with an insurance company. System A comprised 13 clinics and system B comprised 17 (Table 1). Race and ethnicity data were available for patients in system A (19% white, 6% black, 69% Hispanic, 4% other) but not system B.

**Table 1:** Characteristics of healthcare systems selected for retrospective cohort studies, Maricopa County, Arizona

Characteristic	System A	System B
Primary care	Yes	Yes
Subspecialty care	Yes	Yes
No. clinics	13	17
Associated with hospital	Yes (public)	No
Racial and ethnic minorities	Majority	Data not available
Insurance	Many without insurance	Most privately insured

Administrative databases from both systems were screened to identify outpatient visits from January 1, 2003, through December 31, 2004, in which patients were assigned primary or secondary codes from the International Classification of Diseases, 9th revision (ICD-9), beginning with 486 (pneumonia, organism unspecified). Patient charts were selected by simple random sampling for chart review to determine whether patients met inclusion criteria. Patients were included if their initial visit was as an outpatient, they had no history of coccidioidomycosis, and they had CAP as defined by the clinician. Patients were excluded if they were hospitalized or had been residents of a long-term care facility within 14 days of symptom onset. Demographic, clinical, diagnostic data (including *Coccidioides* spp. testing), and outcomes were extracted from medical records for all subsequent visits within 2 months after the initial clinic visit.

Preliminary results, obtained by using Current Procedural Terminology codes for *Coccidioides* spp. serologic testing (86635, 86329, 86331, or 86171), indicated that 13% of patients in system B were being tested for *Coccidioides* spp. compared with 1% in system A. Assuming α = 0.05 and a power of 80%, a sample size of 86 was needed for each cohort to show a difference in testing frequency between the 2 systems.

### Case–Control Study

To determine factors associated with coccidioidomycosis testing (testing predictors) among CAP patients, we conducted a case–control study in system B. We identified all ambulatory patients who had visited system B in 2003 and 2004 and had an ICD-9 code for “pneumonia, organism unspecified” and a Current Procedural Terminology code for *Coccidioides* spp. serologic testing. Patients with CAP were defined by the same criteria used in the cohort studies. Case-patients were defined as CAP patients who had received *Coccidioides* spp. serologic testing, regardless of test result. Controls were defined as patients who met the definition for CAP but had not received *Coccidioides* spp. serologic testing. A simple random sample of visits was reviewed to confirm inclusion criteria and to confirm *Coccidioides* spp. serologic testing associated with the pneumonia.

### Statistical Analysis

All data were entered into Epi Info (Centers for Disease Control and Prevention [CDC], Atlanta, GA, USA). Analysis was performed in Epi Info, SAS version 9.1 (SAS Institute, Inc., Cary, NC, USA), and StatXact 6 (Cytel Software Corp., Cambridge, MA, USA). Confidence intervals (CIs) on difference in proportions were determined by assuming 2 independent binomial proportions and using an exact method. The Kruskal-Wallis test was used to compare continuous variables. Univariate logistic regression models related the dependent variable (a clinician ordering a test for *Coccidioide*s spp.) to independent variables such as age (>18 years vs. <18 years); reported rash; chest pain; symptoms >14 days, as well as other demographic or clinical symptom characteristics. A final multivariable model was chosen by using the stepwise selection procedure. Independent variables that remained significant (α<0.05) while other significant variables were controlled for were included in the final model. Exact 95% CIs are reported because of low observed cell counts.

## Results

### Descriptive Epidemiology

In 2004, Maricopa County had the most reported cases of coccidioidomycosis in Arizona (2,704 cases), followed by Pima and Pinal Counties (715 and 164 cases, respectively). Compared with other counties in Arizona, Maricopa, Pima, and Pinal Counties also had the highest incidence rates (77, 77, and 75 cases per 100,000 persons, respectively). The incidence rate in Maricopa County has increased from 42 per 100,000 persons in 1999 to 77 cases per 100,000 persons in 2004. Maricopa County shows a seasonal pattern with cases peaking in the winter season (Figure), similar to findings reported elsewhere (*9*). Coccidioidomycosis was reported in every 5-year age category; the incidence rate for coccidioidomycosis was 8/100,000 for those <5 years of age and increased steadily with each age group; incidence for persons 55–64 years of age was the highest (166/100,000). Groups of persons >65 years of age also had high incidence rates (65–74 years, 161/100,000; 75–84 years, 147/100,000; >85 years, 153/100,000).

**Figure:**
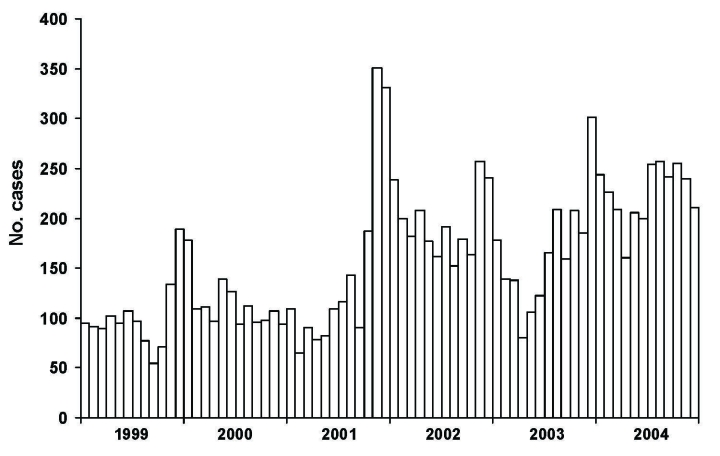
Coccidioidomycosis cases reported by month, Maricopa County, Arizona, 1999–2004.

### Retrospective Cohort Studies

#### Participants

In system A, 619 visits for “pneumonia, organism unspecified” were identified, from which 132 (21%) were sampled for chart review. From the 132, 66 (50%) were excluded: 11 had no charts available, 21 were miscoded or did not have a clinical diagnosis of pneumonia, 30 were initially hospitalized, and 4 had been residents of long-term care facilities or hospitalized within the 14 days before symptom onset. The remaining 66 patients were confirmed by chart review to have CAP and were included.

In system B, 14,695 visits for “pneumonia, organism unspecified” were identified, from which 159 (1%) were sampled for chart review. From the 159, 72 (46%) were excluded: 6 had no charts available, 25 did not have clear documentation of pneumonia, 37 were initially hospitalized, 1 had been hospitalized within 14 days before symptom onset, and 3 had a history of coccidioidomycosis. The remaining 87 patients were therefore included.

**Patient Characteristics and Clinical Description** (Table 2)

**Table 2:** Demographic and clinical characteristics of patients with community-acquired pneumonia included in retrospective cohort studies, Maricopa County, Arizona, January 2003–December 2004*

Patient characteristic	System A (% or range), n = 66	System B (% or range), n = 87	Absolute difference in percentages (95% CI)*
Evaluated initially in emergency department	17 (26)	5 (6)	20 (7–33)†
Median age, y (range)	54 (6–90)	37 (0–86)	
Age <18 y	7 (11)	32 (37)	26 (12–39)†
Male	30 (46)	47 (54)	NS
Median no. days of symptoms before 1st visit	8 (1–30)	7 (1–60)	NS
Symptoms for >21 d at time of 1st visit	4 (9)	6 (7)	NS
Symptoms			
Fever	22 (33)	50 (58)	NS
Chills	5 (8)	12 (14)	NS
Night sweats	0	4 (5)	NS
Myalgias	2 (3)	3 (3)	NS
Fatigue	4 (6)	4 (5)	NS
Cough	54 (82)	69 (79)	NS
Dyspnea	18 (27)	23 (26)	NS
Chest pain	10 (15)	11 (13)	NS
Wheezing	8 (12)	9 (10)	NS
Signs			
Temperature >100.4°F	10 (15)	24 (28)	NS
Tachycardia	8 (12)	11 (13)	NS
Focal lung examination	37 (56)	44 (51)	NS
Hypoxia	0	0	NS
Rash	0	0	NS
Immunosuppressive medication	2 (3)	1 (1)	NS
Coexisting conditions			
Asthma	10 (15)	23 (26)	NS
Chronic obstructive pulmonary disease	13 (20)	12 (14)	NS
Diabetes mellitus	25 (38)	7 (8)	30 (16–43)†
HIV infection	0	1 (1)	NS
Pregnancy	1 (2)	0	NS
Transplant	1 (2)	0	NS
Malignancy	1 (2)	3 (3)	NS
Diagnostic testing, noncoccidioidal			
Chest radiograph	23 (35)	83 (95)	61 (47–72)†
Radiographically proven pneumonia	18 (27)	67 (83)	50 (35–63)†
Treatment and outcome			
Antibacterial drugs	66 (100)	87 (100)	NS
Follow-up visits			
None	17 (26)	27 (31)	NS
1	34 (52)	27 (31)	NS
2	5 (8)	13 (15)	NS
>3	10 (15)	20 (23)	NS
Hospital admissions	2 (3)	6 (7)	NS
Died	0 (0)	2 (2)	NS
*Coccidioides* spp. serologic testing			
At any visit	1 (2)	11 (13)	11 (3–20)‡
During follow-up visit	0	4 (5)	NS
Reactive results	0	1 (1)	NS
Median no. days before testing	12	27 (1–99)	–
Symptoms >14 days before testing	0	7 (64)	NS
Diagnosis of coccidioidomycosis, any technique	0	1 (1)	NS

CI, confidence interval; NS, not significant. CIs on difference in percentages were performed by using an exact method with 2 independent binomial proportions. †p<0.01. ‡p<0.05.

Of CAP patients, 11% of those in system A were <18 years of age compared with 37% in system B (difference 26%, 95% CI 12%–39%, p<0.01). Patients in system A were significantly more likely than patients in system B to have been seen initially in an emergency department (26% vs. 6%, difference 20%, 95% CI 7%–33%, p<0.01).

Clinical signs and duration of symptoms before the initial visit were similar for patients in both systems. In systems A and B, respectively, cough was the most commonly reported symptom (82% and 79%), followed by fever (33% and 58%) and dyspnea (27% and 26%). Approximately 50% of patients in each group had focal findings on lung examination.

In terms of coexisting conditions, 38% of patients in system A had diabetes compared with 8% in system B (difference 30%, 95% CI 16%–43%, p<0.01); diabetes is a risk factor for severe pneumonia and for complicated *Coccidioides* spp. infection (*10*). Similar proportions of those with asthma or chronic obstructive pulmonary disease were found in both systems. Few patients had a diagnosis of HIV, pregnancy, or organ transplantation, and few were receiving immunosuppressive medications.

Chest radiographs were less likely to have been taken for patients in system A than for patients in system B (23% vs. 83%, difference 60%, 95% CI 47%–72%, p<0.01). Of those in both systems who did have chest radiographs taken, similar proportions had findings consistent with pneumonia (78% and 81%, respectively). All patients in both systems received treatment with antibacterial drugs.

*Coccidioides* spp. serologic testing was low overall for patients in systems A (1/66, 2%, 95% CI 0.04%–8%) and B (11/87, 13%, 6%–22%). Patients in system A were significantly less likely than patients in system B to have had *Coccidioides* spp. serologic testing performed at any point during the clinical course of disease (difference 11%, 95% CI 3%–20%, p = 0.01). In system B, 7 (64%) of 11 tested patients were tested during a follow-up visit rather than at the initial visit. Of those 11 patients, 1 (9%) had a reactive serologic test result for *Coccidioides* spp. Median duration of symptoms before testing for patients in system B was 27 days (range 1–99 days); most patients (64%) were tested after symptoms had been present for at least 2 weeks.

Patient outcomes were similar. In system A, 2 (3%) were known to have been hospitalized for worsening of pneumonia, and no patients were known to have died. In system B, 6 patients (7%) were hospitalized for worsening pneumonia, and 2 died; cause-of-death data were unavailable.

### Case–Control Study

Of 14,695 potential CAP-patient visits in system B, we randomly selected 60 case-patients (i.e., those with CAP who had been tested for *Coccidioides* spp.) and 76 controls (i.e., those with CAP who had not been tested). According to univariate analysis, patients who had chest pain (odds ratio [OR] 4.6, 95% CI 1.8–11.8), rash (OR undefined, 95% CI 1.2–undefined), or those with symptom duration >14 days (OR 5.8, 95% CI 2.1–15.7) were significantly more likely to have been tested (Table 3). Additionally, patients >18 years of age (OR 5.5, 95% CI 2.1–15.3) and those who had diabetes or were receiving an immunosuppressive medication (OR 3.6, 95% CI 1.0–16.5) were significantly more likely to have been tested.

**Table 3:** Characteristics of patients with community-acquired pneumonia (CAP) who were tested for coccidioidomycosis, Maricopa County, Arizona, January 2003–December 2004*

Characteristic	Case-patients, no. (%), n = 60	Controls, no. (%), n = 76	Odds ratio (95% CI)	Adjusted odds ratio† (95% CI)
Age >18 y	53 (88)	44 (58)	5.5 (2.1–15.3)‡	5.3 (1.5–24.0)
Male	28 (55)	40 (53)	0.9 (0.5–1.9)	NS
Chest pain	19 (32)	7 (9)	4.6 (1.8–11.8)‡	3.9 (1.2–13.8)
Rash	5 (8)	0	Undefined (1.2–Undefined)‡	21.1§ (2.2–undefined)
Diabetes mellitus or immunosuppressive condition	10 (17)	4 (5)	3.6 (1.0–16.5)‡	NS
Symptoms >14 d	20 (33)	6 (8)	5.8 (2.1–15.7)‡	4.1 (1.3–14.2)

*CI, confidence interval; NS, not significant. Case-patients were patients who had CAP and had received *Coccidioides* spp. serologic testing, regardless of test result; controls were patients who had CAP but had not received *Coccidioides* spp. serologic testing. †Adjusted odds ratios and exact 95%CI from a multivariable logistic regression model. ‡Significant (p<0.05) according to univariate analysis. §Median unbiased estimate of the adjusted odds ratio.

The multivariate model identified the following as being significantly more likely to have been tested for coccidioidomycosis: adult patients (adjusted OR 5.3, 95% CI 1.5–24.0) and those who reported rash (adjusted OR 21.1, 95% CI 2.2–∞), chest pain (adjusted OR 3.9, 95% CI 1.2–13.8), or symptoms for >14 days (adjusted OR 4.1, 95% CI 1.3–14.2). The Hosmer-Lemeshow goodness-of-fit test showed no evidence of a lack of fit (p = 0.8).

Of the 60 case-patients who were tested for *Coccidioides* spp., 9 (15%, 95% CI 8%–26%) had positive results. Of these 9, 3 had immunoglobulin (Ig) M by enzyme immunoassay alone, 3 had IgM and IgG by enzyme immunoassay (IgG titers 4, 8, and 8 by complement fixation), 1 had IgM and IgG by immunodiffusion, 1 had a single high IgG titer (128), and another had a rising IgG titer (<2 initial; 16 at 4 weeks).

## Discussion

Our study directly measured serologic testing practices for coccidioidomycosis. The proportion of ambulatory patients with CAP who were tested for *Coccidioides* spp. was low in this coccidioidomycosis-endemic area.

Because incidence rates of CAP in this area of the United States are not available, the number of CAP patients who do not receive serologic testing for *Coccidioides* spp. cannot be estimated. However, if incidence rates are comparable to those in other parts of the country (*6*), the number of patients with CAP who are not tested for coccidioidomycosis would be high. If CAP is the result of coccidioidomycosis in as many as 10%–15% of these untested patients, then large numbers of patients would remain undiagnosed.

According to recently published Infectious Disease Society of America (IDSA) guidelines, the benefit of antifungal therapy for uncomplicated respiratory *Coccidioides* spp. infection is unknown (*6*). However, treatment is more likely to benefit groups at risk for severe or disseminated infection (*6*). Although these groups are especially likely to benefit from early testing for coccidioidomycosis, other benefits of early diagnosis may exist for all patients with coccidioidomycosis, regardless of risk for severe disease. Such benefits may include avoidance of unnecessary use of antibacterial agents, earlier identification of complications, decreased need for further expensive diagnostic studies, and reduction of patient anxiety (*3*).

Reasons that CAP patients may not be tested for *Coccidioides* spp. are unclear but are likely complex. First, professional consensus for optimal testing practices may have been lacking. Although guidelines developed by national professional organizations for the management of CAP were available during the study period (*11*–*13*), these guidelines did not directly address the best strategy for *Coccidioides* spp. serologic testing (*11*–*13*). IDSA guidelines for the treatment of coccidioidomycosis also did not clearly recommend serologic testing for patients with CAP (*6*,*14*). The most recent IDSA/American Thoracic Society guidelines for CAP, published after the study period, now recommend evaluating travel history or exposure to disease-endemic area during the initial assessment rather than waiting for a failed response to therapy (*15*); these guidelines may lead to increased testing for coccidioidal CAP. Second, physicians may be unaware of the benefits of early testing or the possible high prevalence of coccidioidomycosis in those with CAP in Arizona and therefore may not understand the utility of testing. Regardless of the reasons, the lack of testing in the presence of widespread disease hampers epidemiologic understanding of this disease and subsequently may affect public health decisions related to resource allocation to control disease, educate physicians, and develop a vaccine.

Our data also illustrate the marked differences in process of care for ambulatory patients with CAP in different health systems. Patients in the uninsured population, system A, were less likely to be tested than patients in the primarily insured population, system B. This disparity is evidenced by the higher proportion of CAP patients in the insured system who received chest radiography in addition to serologic testing. Public health officials may be able to address these disparities by providing general recommendations for diagnostic testing of patients with CAP in coccidioidomycosis-endemic areas; process-of-care measures such as chest radiography and *Coccidioides* spp. serologic testing may help determine effectiveness of such interventions.

Of the tested CAP patients in system B, 15% had serologic evidence of recent *Coccidioides* spp. infection; this proportion is much lower than that (29%) found in a recently reported study (*8*). Several differences may explain this discrepancy. First, our study population was located in a different area of Arizona. Second, our definition of CAP differed from the definition used in the other study and is likely more representative of actual CAP found in outpatient practices (*8*). However, our proportion may overestimate the true proportion of CAP caused by coccidioidomycosis because testing in our cohort was subject to a decision made by the treating physician. Nevertheless, the high proportion could signify that a large number of pulmonary coccidioidomycosis diagnoses are likely missed in Maricopa County alone and that the overall extent of pulmonary coccidioidomycosis is higher than that indicated by reportable disease data. Further studies are needed to better quantify the extent of disease.

Our study has several limitations. First, in contrast to definitions used in many studies, our definition of CAP included patients whose diagnosis was made by a clinician without a chest radiograph or with a negative chest radiograph. However, although some patients may not have truly had CAP, our definition reflects what clinicians actually believed they were treating, which is clinically relevant to whether a diagnostic test is ordered. Second, although the study populations were geographically dispersed throughout metropolitan Phoenix and included varied population segments, they may not be generalizable to populations in health systems in other areas of Arizona. Third, at system B, controls were inadvertently oversampled during 2003, so we were unable to include visit year in our analysis. However, it is unlikely that the biased selection based on year led to substantial bias for other variables such as age, sex, clinic location, signs, symptoms, coexisting medical conditions, or testing. Fourth, data on socioeconomic status were not available for either system, and race or ethnicity data were not available from system B. Fifth, because our study evaluated ambulatory rather than hospitalized patients with CAP, our conclusions cannot be generalized to the hospital, where testing practices are likely to differ.

Our study shows that testing for *Coccidioides* spp. among ambulatory patients with CAP is infrequent in metropolitan Phoenix. Providers in metropolitan Phoenix and other coccidioidomycosis-endemic areas should consider testing patients with CAP for coccidioidomycosis. Further epidemiologic studies are needed to better determine the true extent of pulmonary coccidioidomycosis.
